# IFN type III: *in vivo* NK cell response

**DOI:** 10.18632/oncotarget.4758

**Published:** 2015-07-04

**Authors:** Fernando Souza-Fonseca-Guimaraes, Arabella Young, Mark J. Smyth

**Affiliations:** Immunology in Cancer and Infection Laboratory, QIMR Berghofer Medical Research Institute, Herston, Queensland, Australia

**Keywords:** Immunology and Microbiology Section, Immune response, Immunity

Despite extensive studies over the last decade reinforcing the role of the immune system in controlling cancer progression and inflammatory disorders, our knowledge of the innate immune system remains quite poor. Natural killer (NK) cells are naturally circulating innate lymphocytes that protect against both tumor development and infection. NK cells exert two major effector functions, the cytotoxic clearance of abnormal target cells and the ability to heighten inflammatory responses through production of cytokines and chemokines. Both mouse and human NK cells exist in at least three differentiation stages [defined by marker pairings such as CD27/CD11b (mouse), DNAM-1+/− (mouse) and CD16/CD56 (human)] [1, 2, 3]. Each of these stages display receptor expression variance associated with their tissue distribution, regulation, survival, cytotoxicity and cytokine/chemokine producing capacity. The activity of NK cells depends on the interplay between a multitude of inhibitory receptors (that bind major histocompatibility complex (MHC) class I molecules) and activating receptors (e.g. NKG2D, CD16 etc.), and they operate in concert to control NK cell effector functions [4].

**Figure 1:**
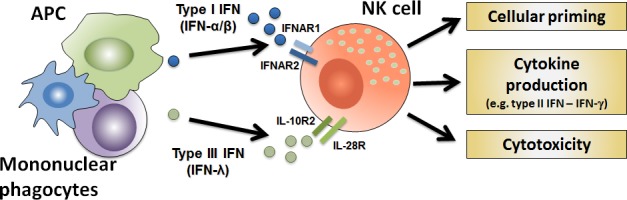
NK cell optimal *in vivo* activity requires type I and III IFN receptors Antigen-presenting cells (APC) and mononuclear phagocytes produce cytokines such as IFN type I and III, which prime NK cells and enhance their effector function for enhanced cytotoxic killing and cytokine production.

Alongside receptor modulation of NK cell function, certain cytokines initiate and maintain NK cell responses. In particular, the interferon family, consisting of three distinct subtypes - type I, II and III IFN, has been associated with improving responses to viral and pathogenic infection. However, regulation of their induction and the distribution of their respective IFN receptors vary, resulting in modulation of function. Type I IFN (IFN-α/β), predominantly produced by activated myeloid cells (e.g. mononuclear phagocytes and dendritic cells), plays an essential role in NK cell priming allowing for optimal cytokine production, cytotoxic killing and antiviral immunity [5] (Figure 1). In response to NK cell priming by type I IFN, production of type II IFN (IFN-γ) is enabled (Figure 1). This cytokine stimulates the adaptive immune response through Th1 polarization of CD4^+^ T cells, which subsequently activates cytotoxic T lymphocytes to initiate aberrant cell clearance [6].

While activation of hematopoietic cells by type I IFN and type II IFN is well established, the role of type III IFN (IFN-λ) in modulating immune cell functions has not been thoroughly explored. The most recently described member of the IFN family, type III IFN shares common anti-viral functions and activation of JAK-STAT signal transduction with type I IFN. However, responses to type III IFN are more limited, due to restricted receptor expression [7]. Recognition of type III IFN occurs through a heterodimer formed by IL-28R and IL-10R2 subunits predominantly found on plasmacytoid DCs, B cells, epithelial cells, and hepatocytes [7]. While the receptor range is limited, IFN-λ has displayed potent anti-viral and anti-tumor responses both via targeting infected or transformed cells directly as well as host-dependent mechanisms.

Recently, using type III IFN receptor (IL-28R^−/−^) gene-targeted mice, we observed that this receptor is involved in the *in vivo* priming of NK cells to enhance their effector functions [8]. Specifically, loss of type III IFN signaling resulted in reduced pro-inflammatory response and improved survival in both lipopolysaccharide (LPS)-induced endotoxicosis and cecal ligation and puncture (CLP)-induced septic shock models. Reduced IFN-γ production by IL-28R-deficient NK cells in response to LPS-induced endotoxicosis was identified in complete gene deletion and NK cell reconstituted immunodeficient Rag2^−/−^γc^−/−^ mice. This indicates that type III IFN contributes to optimal activation of NK cells, enabling initiation of IFN-γ production (Figure 1). In addition, reduced NK cell antitumor activity was evident in several transplantable and spontaneous cancer models in IL-28R-deficient compared to wild type mice. While we detected IL-28R mRNA by RT-PCR, we were unable to demonstrate *in vitro* that pegylated IFN-λ (PEG-IL-28A) acts on NK cell cytokine production or killing. In contrast, *in vivo* treatment with PEG-IL-28A enhanced antitumor responses from wild type NK cells both alone and in combination with type I IFN [8].

Currently, targeting NK cells via immunotherapies to bolster their activity is of major interest, due to their rapid antigen-independent host immune response. Here, we identified that NK cells require IL-28R signaling for optimal *in vivo* activity [8]. Both IFN-λ and IFN-α have been utilized clinically as anti-viral therapies, as well as anti-tumor treatments for the latter. While IFN-α displays high toxicity, due to widespread receptor expression, the restricted range of IFN-λ leads to improved safety profiles. Therefore, utilization of IFN-λ as an adjuvant therapy to achieve optimal NK cell effector function should be considered as a potential combinatorial treatment modality.
